# Correlation between vestibular response to caloric stimulation and cochlear function in Ménière's disease

**DOI:** 10.1016/j.bjorl.2024.101513

**Published:** 2024-09-19

**Authors:** da Rosa Heinen Leticia, Assis Moro da Rocha Filho Marcelo, Zanelatto Santos Artur, Calage Pinto Amanda, Afonso de Freitas Toledo Samuel, Lavinsky Luiz, Lavinsky Joel

**Affiliations:** a Universidade Federal do Rio Grande do Sul (UFRGS), Programa de Pós-Graduação em Medicina: Ciências Cirúrgicas, Porto Alegre, RS, Brasil; b Universidade Federal do Rio Grande do Sul (UFRGS), Porto Alegre, RS, Brasil; c Universidade Federal do Rio Grande do Sul (UFRGS), Faculdade de Medicina, Porto Alegre, RS, Brasil

**Keywords:** Ménière's disease, Caloric test, Dizziness, Pure-tone audiometry, Hearing loss, Sensorineural

## Abstract

**Objective:**

To analyze the correlation between caloric testing response patterns with respect to cochlear impairment in individuals with unilateral Ménière's disease.

**Methods:**

In this observational cross-sectional study, the factor under study was Ménière's disease and the outcome was cochleovestibular function, evaluated through caloric stimulation using videonystagmography and four-frequency averages in pure tone audiometry.

**Results:**

A total of 187 patients (equal sex distribution) who met the inclusion criteria were included. In impairment analysis of the affected ear, 17 patients had only vestibular impairment, 56 had only cochlear impairment, and 114 had cochleovestibular impairment. The distribution of Ménière's disease stages according to four-frequency average was grade I: 23.53%, grade II: 16.04%, grade III: 42.25%, and grade IV: 18.18%. There was a significant association (*p* < 0.001) between the affected ear and labyrinthine preponderance. The Spearman correlation between four-frequency average and labyrinth preponderance (*r* = 0.326) indicated a moderate correlation, considering *p* < 0.01.

**Conclusion:**

Our results show that the greater the cochlear damage due to Ménière's disease, the more impaired the vestibular function in the affected ear.

**Level of evidence:**

Level 2 - Individual cross-sectional studies with consistently applied reference standard and blinding

## Highlights

• The study has a moderate correlation of the caloric test and four-frequency average.
• Between grades I and II, there was a greater impact on caloric tests in grade I.
• The cochlear damage was directly proportional to the vestibular deficit.

## Introduction

In 1861, Prosper Ménière found the inner ear to be the site of injury in several patients who shared hearing disorders and vertigo.^1^ Considered a degenerative disease of the inner ear, Ménière's Disease (MD) is characterized by vertigo, tinnitus, and hearing loss, which may or may not be accompanied by a feeling of aural fullness. Many etiological factors are correlated with MD, including metabolic changes, due to the fact that the labyrinthine structures are sensitive to changes in glucose and oxygen levels.^2^

According to more current criteria by the Bárány Society, MD diagnosis is based on clinical and audiometric factors, with sensorineural hearing loss being a necessary symptom.^2^ Normally, a patient's hearing fluctuates throughout MD progression and, during the initial stages, the patient may completely recover after a crisis.^3^ In 1995, the American Academy of Otolaryngology-Head and Neck Surgery (AAO-HNS) outlined the clinical course of MD according to four-frequency average of pure-tone auditory thresholds (500, 1000, 2000, and 3000 Hz): grade 1 (≤25 dB), grade 2 (26–40 dB), grade 3 (41–70 dB) and grade 4 (>70 dB).^2^

Studies have identified a prevalence of cochlear symptoms in MD, such as hearing loss (87.7%) and tinnitus (91.1%).^4^ Paparella et al. demonstrated that the most common audiometric pattern is in the form of a “peak” (compromising low and high frequencies while preserving medium frequencies).^5^

MD seems to present with a predominance of vestibular or auditory symptoms. Studies have observed that 50% of patients have vertigo and hearing loss, while 19% and 26% have only vertigo or hearing loss, respectively. Due to these variations, terms such as vestibular Ménière’s disease and cochlear Ménière’s disease are used.^6^

Considered the gold standard for assessing and diagnosing asymmetric and unilateral changes, because it allows for separate assessments, caloric stimulation is extremely important for MD evaluation.^7^

Evaluating the lateral semicircular canals, caloric stimulation reveals no pathognomonic findings of MD. Studies show variable results, the most common being hyporeflexia of the affected labyrinth.^8^ About 75% of patients with unilateral MD have a hypofunctional response on the affected side. However, in other studies more than 50% of the patients have a normal response. Due to different stages in the clinical course of MD, the results of caloric stimulation vary greatly in different studies.^2^

Although studies have already proven that MD causes cochlear damage, which leads to hearing loss and dizziness, this disease is still diagnosed according to clinical symptoms, especially in the initial stages. Therefore, this study aimed to evaluate the correlation between vestibular response to caloric stimulation and cochlear function in patients with unilateral MD.

## Methods

This observational cross-sectional study with a convenience sample reviewed the medical records of 1328 patients with suspected MD who were evaluated at the otorhinolaryngology and speech therapy service of a private clinic in the city of Porto Alegre, Brazil between 2008 and 2020. Of these patients, 187 fulfilled the eligibility criteria and were included.

The 187 patients’ auditory tonal thresholds and caloric test outcomes were collected without the examiner’s knowledge. The tests were conducted at different times and analyzed separately in order to keep the variables blind.

We included patients who had been clinically diagnosed with unilateral MD and were evaluated by an otorhinolaryngologist based on Bárany Society criteria. The otorhinolaryngologist also performed the pure-tone audiometry examinations and the caloric stimulation. The study was approved by the institutional review board (protocol 4.362.864) and conducted in accordance with Resolution 466/12. Patients with bilateral MD or those with “Ménière-like” symptoms (e.g., acoustic neuroma, multiple sclerosis, neurosyphilis, vestibular migraine, perilymphatic fistula, otosclerosis, superior semicircular canal dehiscence, etc.) were excluded.^9^

Based on the data analysis protocol, patients were classified according to the stage of MD using the four-frequency average of pure-tone auditory thresholds (500, 1000, 2000, and 3000 Hz): grade 1 (≤25 dB), grade 2 (26–40 dB), grade 3 (41–70 dB), and grade 4 (>70 dB).^7^ In a secondary analysis of the same data, caloric stimulation was classified using Jongkees’ formula:

$$\text{L}\text{P}\;=\;\frac{\left(\text{S}\text{C}\text{A}\text{V}\;50{}^{\circ}\;\text{R}\text{E}+\text{S}\text{C}\text{A}\text{V}\;24{}^{\circ}\;\text{R}\text{E})-(\text{S}\text{C}\text{A}\text{V}\;50{}^{\circ}\;\text{L}\text{E}\;+\;\text{S}\text{C}\text{A}\text{V}\;24{}^{\circ}\;\text{L}\text{E}\right)}{\left(\text{S}\text{C}\text{A}\text{V}\;50{}^{\circ}\;\text{R}\text{E}+\text{S}\text{C}\text{A}\text{V}\;24{}^{\circ}\;\text{R}\text{E})\;+\;(\text{S}\text{C}\text{A}\text{V}\;50{}^{\circ}\;\text{L}\text{E}+\text{S}\text{C}\text{A}\text{V}\;24{}^{\circ}\;\text{L}\text{E}\right)}x100$$

where: LP, Labyrinthine preponderance; SCAV, Slow Component Angular Velocity; RE, Right Ear; LE, Left Ear.

The main objective of pure-tone audiometry is to determine the integrity of the auditory system, in addition to identifying the type, degree, and configuration of hearing loss in each ear, determining the frequency in Hz and the intensity in dB. The intensity level is between 0 and 120 or 125 dB (Hearing Level) HL, depending on the device’s maximum output, for air conduction thresholds, and between 50 and 65 dB HL for bone conduction thresholds. The minimum intensity at which the patient can detect a pure tone at each frequency evaluated in air (250, 500, 1000, 2000, 3000, 4000, 6000, and 8000 Hz) or bone conduction (500, 1000, 2000, 3000, and 4000 Hz) is determined in both the Right Ear (RE) and Left Ear (LE). This test is considered the gold standard for hearing assessment, since it can determine hearing impairment and the topical diagnosis of the lesion, ie, outer, middle, and/or inner ear.^10^

The examinations were performed in an acoustic booth using an Interacoustics AC30 Clinical Audiometer (Interacoustics A/S, Middelfart, Denmark) and Telephonics TDH-39 supra-aural headphones (Griffon, New York, NY, USA). The results were transferred to a computer using PersonalMed 98 (Totvs, São Paulo, SP, Brazil), resulting in a graph of the audiological results.

Caloric stimulation, a component of videonystagmography, was performed using an ICS Chartr 200 testing system and an ICS AirCal irrigator (GN Otometrics A/S, Copenhagen, Denmark) at air temperatures of 24 °C and 50 °C.

After recording the cold (24 °) and hot (50 °) stimulation results for the RE and LE, Jongkees’ formula was used to verify the results, comparing values corresponding to the ear (labyrinthine preponderance) or the direction of nystagmus beats (directional preponderance). Indices <20% for labyrinthine preponderance and <30% for directional preponderance are considered normal.

The inclusion process involved labyrinthine preponderance analysis in each patient with unilateral MD.

After completing the recordings, the labyrinthine preponderance was calculated in PersonalMed 98. IBM SPSS Statistics 20.0 (IBM, Armonk, NY, USA) was used to analyze the computed data. Normally distributed continuous variables were expressed as mean and SD. Categorical variables were expressed as percentages. Spearman’s correlation coefficient was used to correlate the MD grade findings (audiometric assessment) with the labyrinth preponderance values (caloric stimulation), and correlations were classified according to Spearman’s coefficient as weak (<0.3), moderate (0.3‒0.5), or strong (>0.5).^11^ The significance level was set at *p* < 0.01.

## Results

Of the 1328 patients with suspected MD, 187 were included in the present study. The main reasons for exclusion were incomplete examinations, bilateral MD, and other concomitant otological pathologies.

Statistical analysis verified practically equally distribution between sex and affected ear, with a mean age of 50 years (Table 1).

**Table 1:** Frequency of sex, age, and affected ear.

Variables	Sex		Age			Affected ear	
n (%) or mean (SD)	Female	Male	Mean	Min	Max	Right	Left
	51.9, n = 97	48.1, n = 90	50.7 y	16 y	79 y	44.9, n = 84	54.1, n = 103
Total	187						

y, years.

Using the four-frequency average (500, 1000, 2000, and 3000 Hz) in pure-tone audiometry, we classified the patients into 4 grades of MD severity based on AAO-HNS definitions. The highest frequency was grade III (42.25%), followed by grade I (23.53%), grade IV (18.18%), and grade II (16.04%). Regarding caloric stimulation response, 33.2% of the patients presented normoreflexia, 36.4% labyrinth preponderance to the right, and 30.5% with labyrinth preponderance to the left.

The association between the affected ear, identified by clinical diagnosis, and caloric stimulation results was significant (*p* < 0.001) according to the Chi-Square test, i.e., those who’s the right ear is affected tend to have a labyrinthine preponderance in the left ear, and those whose left ear affected tend to have a labyrinth preponderance in the right ear (Table 2).

**Table 2:** Association of the affected ear with MD with the results of caloric stimulation.

Caloric stimulation	Right ear affected (%)	Left ear affected (%)
Normoreflexia	32.1	34.1
LP to the right	2.4	64.1
LP to the left	65.5	1.9

LP, Labyrinthine Preponderance.

The response to caloric stimulation and pure-tone audiometry using the four-frequency average (500, 1000, 2000, and 3000 Hz) was correlated with each MD grade according to the AAO-HNS. In grade I, the initial stage of MD, the four-frequency average decibel Hearing Level (dB HL) is ≤25, and 56.8% of these patients had labyrinthine preponderance. Grade II (26–40 dB HL) had the lowest rate of labyrinthine preponderance: 43.3%. Grades III (41–70 dB HL) and IV (>70 dB HL), which involve greater cochlear impairment, had the highest labyrinthine preponderance: 73.4% and 85.3%, respectively.

**Fig. 1:**
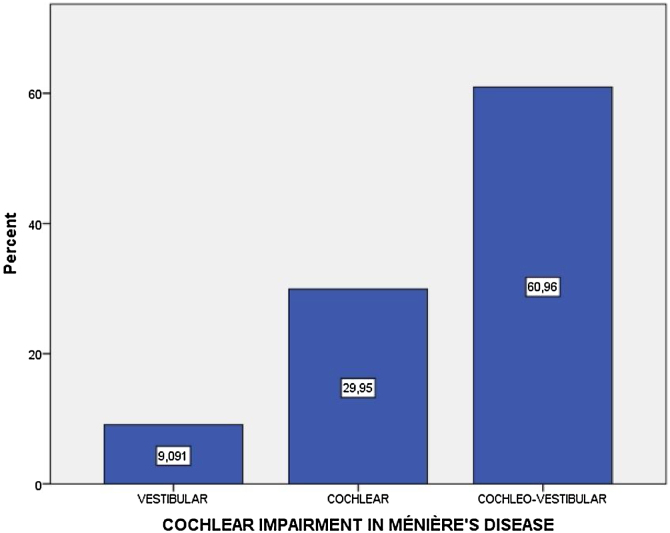
Percentage of individuals with cochlear, vestibular, and cochleovestibular impairment in unilateral MD.

To assess the degree of cochlear impairment that MD can cause in the affected ear, we separated the 187 patients into those with only vestibular impairment, only cochlear impairment, and those with cochleovestibular impairment; the latter group was the largest (Fig. 1).

**Fig. 2:**
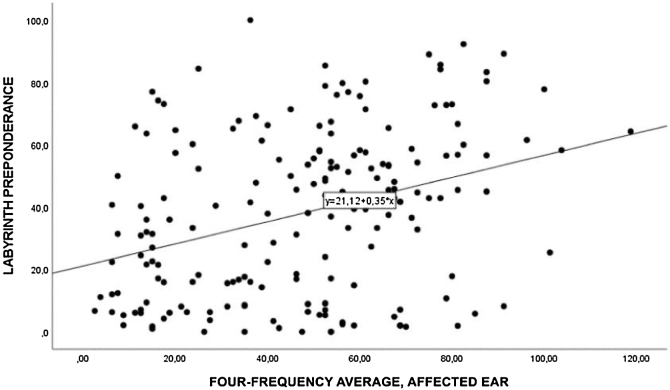
Spearman's correlation between the four-frequency average in the affected ear and labyrinth preponderance.

The Spearman correlation between four-frequency average and labyrinth preponderance showed a moderate correlation (*r* = 0.326), considering *p* < 0.01. This demonstrates the presence of a correlation between pure-tone audiometry grades and labyrinth preponderance: the greater the cochlear damage on pure-tone audiometry, the more impaired the vestibular function in the affected ear (Fig. 2).

**Fig. 3:**
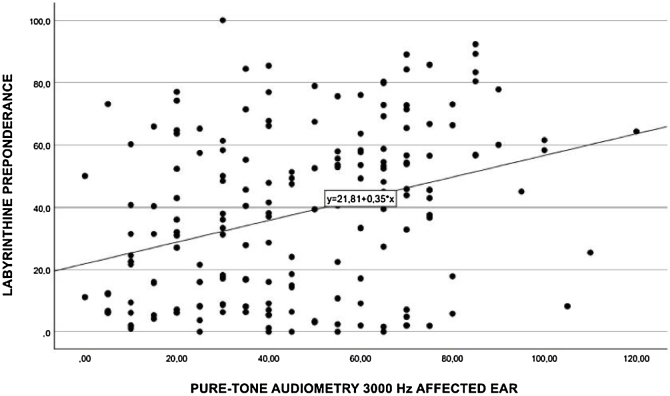
Spearman's correlation of labyrinthine preponderance at a frequency of 3000 Hz.

**Fig. 4:**
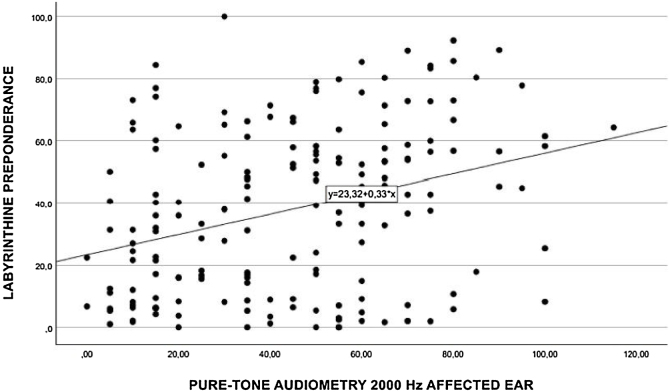
Spearman's correlation of labyrinthine preponderance at a frequency of 2000 Hz.

**Fig. 5:**
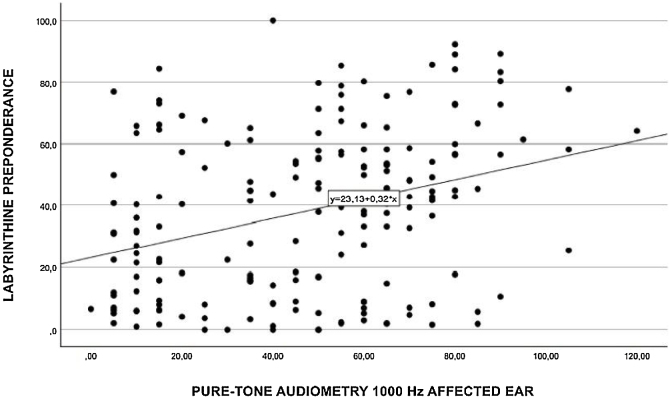
Spearman's correlation of labyrinthine preponderance at a frequency of 1000 Hz.

**Fig. 6:**
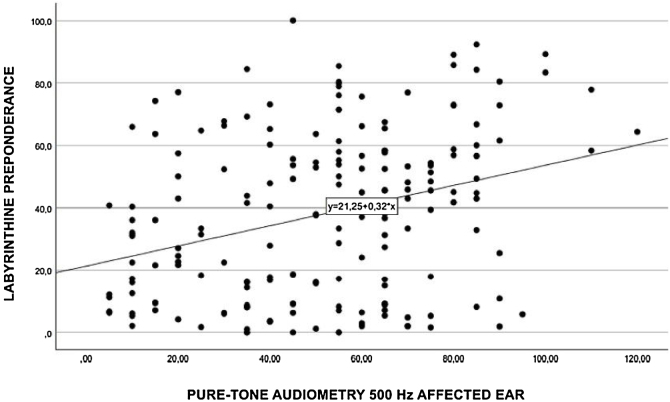
Spearman's correlation of labyrinthine preponderance at a frequency of 500 Hz.

Performing a separate Spearman’s correlation of labyrinth preponderance at each individual frequency, moderate associations (Fig. 3) were found at 3000 Hz (*p* = 0.330), 2000 Hz (*p* = 0.315) (Fig. 4), 1000 Hz (*p* = 0.322) (Fig. 5), and 500 Hz (*p* = 0.29) (Fig. 6).

## Discussion

According to AAO-HNS guidelines, MD classification in clinical studies must be performed objectively through the four-frequency average, since the number of vertigo attacks and the intensity of tinnitus/aural fullness are extremely subjective and ambiguous.^12^

Over the years, the clinical criteria for diagnosing MD have improved, and an instrumental profile has generated great interest in the area. Defining an instrumental profile of MD guarantees an objective method of identification and characterization of the disease, allowing a diagnosis in the initial stages.

Studies have shown that vestibular assessment with caloric stimulation can help with MD diagnosis. A retrospective study of 72 patients with unilateral MD found that patients with MD have abnormalities in caloric vestibular test results and, in the final stages, after the onset of the cochlear impairment, the abnormalities increase.^13^

A 2017 study of patients with unilateral MD analyzed canal paresis found a correlation between decline in objective hearing and horizontal semicircular canal function in caloric vestibular testing, with a 1:1 ratio indicating differential involvement of both systems.^14^

In a study of 78 patients with unilateral MD, Sluydts et al.^15^ found a significant correlation (*p* = 0.036) when cochlear impairment was present in grade II. There was no significant difference in the correlations between pure tone audiometry and the vestibular examinations cervical vestibular evoked myogenic potentials and Video Head Impulse Test (vHIT). In caloric stimulation, they found that the greater the cochlear impairment in pure tone audiometry, the greater the vestibular hypofunction of the affected ear.

Another study comparing the response to caloric stimulation with the vHIT test in MD patients found that 76.5% had vestibular hypofunction of the affected ear in caloric stimulation. On the other hand, vHIT detected altered vestibular function in only 47.1% of the same sample, showing that caloric stimulation is more sensitive to vestibular function changes in all stages of MD.^16^

Rubin et al.^17^ conducted a study of 37 patients diagnosed with unilateral MD, comparing vHIT and caloric stimulation. Although all patients had normal responses in vHIT, 45% had an abnormal response in caloric stimulation.

In a literature review (PubMed, LILACS and SciELO), we found that despite the existence of several studies comparing examinations in MD, the samples were relatively small, thus more reliable results are necessary. In the present study, we managed a relatively large number of patients (n = 187), which allowed us to determine the effectiveness of caloric stimulation in relation to cochlear impairment.

The present study clearly demonstrated that progressive cochlear impairment in the inner ear manifested itself with progressively reduced labyrinthine function on the affected side, which agrees with recent studies associating tonal audiometry and vestibular canal paresis response.

Although caloric stimulation does not help diagnose MD, its importance in determining cochleovestibular impairment in the affected ear is indisputable, helping determine the affected side, thus allowing early targeted treatment.

## Conclusion

Therefore, despite great variability due to the asymmetric distribution, we can say that caloric stimulation can help classify vestibular impairment, and the greater the cochlear damage due to MD, the greater the labyrinthine function deficit in that ear.

## Ethical approval

The study was approved by the institutional review board (protocol 4.362.864) and conducted in accordance with Resolution 466/12 of the Brazilian Ministry of Health’s National Health Council and the Declaration of Helsinki.

## Informed consent

Not applicable.

## Financial disclosure

This research did not receive any specific grant from funding agencies in the public, commercial, or not-for-profit sectors.

## Conflicts of interest

The authors declare no conflicts of interest.
